# 2-(5,6-Dihydro­benzimidazo[1,2-*c*]quinazolin-6-yl)-5-methyl­phenol

**DOI:** 10.1107/S1600536811032673

**Published:** 2011-08-27

**Authors:** Naser Eltaher Eltayeb, Siang Guan Teoh, Madhukar Hemamalini, Hoong-Kun Fun

**Affiliations:** a School of Chemical Sciences, Universiti Sains Malaysia, 11800 USM, Penang, Malaysia; b Department of Chemistry, Faculty of Pure and Applied Sciences, International University of Africa, Sudan; c X-ray Crystallography Unit, School of Physics, Universiti Sains Malaysia, 11800 USM, Penang, Malaysia

## Abstract

In the title compound, C_21_H_17_N_3_O, the imidazole ring is essentially planar, with a maximum deviation of 0.009 (1) Å. The mol­ecule is disordered over two sites corresponding to a rotation of approximately 180° with a refined occupancy ratio of 0.9180 (14):0.0820 (14). The central pyrim­idine ring makes dihedral angles of 5.02 (5), 3.97 (5) and 6.28 (5)°, respectively, with the planes of the imidazole and the terminal phenyl rings for the major component; the values for the minor component are 5.8 (7), 5.0 (6) and 8.5 (6)°, respectively. Part of the observed planarity is accounted for in terms of an intra­molecular N—H⋯O hydrogen bond. In the crystal, mol­ecules of the major component are connected by O—H⋯N hydrogen bonds, forming supra­molecular chains along the *c* axis.

## Related literature

For applications of benzimidazoles, see: Sun *et al.* (2010 ▶); Harrell *et al.* (2004 ▶). For related structures, see: Eltayeb *et al.* (2007 ▶, 2009 ▶, 2011 ▶). For the stability of the temperature controller used in the data collection, see: Cosier & Glazer (1986 ▶).
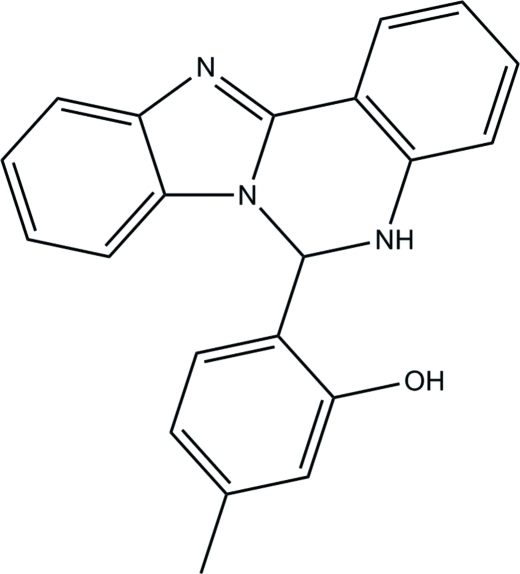


## Experimental

### 

#### Crystal data

C_21_H_17_N_3_O
*M* *_r_* = 327.38
Monoclinic, 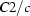

*a* = 15.1292 (3) Å
*b* = 12.2648 (2) Å
*c* = 17.1909 (3) Å
β = 96.233 (1)°
*V* = 3171.03 (10) Å^3^
*Z* = 8
Mo *K*α radiation
μ = 0.09 mm^−1^
*T* = 100 K
0.59 × 0.21 × 0.20 mm

#### Data collection

Bruker SMART APEXII CCD area-detector diffractometer
Absorption correction: multi-scan (*SADABS*; Bruker, 2009 ▶) *T*_min_ = 0.951, *T*_max_ = 0.983
24277 measured reflections
6164 independent reflections
4808 reflections with *I* > 2σ(*I*)
*R*_int_ = 0.029

#### Refinement

*R*[*F* ^2^ > 2σ(*F* ^2^)] = 0.045
*wR*(*F* ^2^) = 0.126
*S* = 1.04
6164 reflections
296 parameters
44 restraints
H atoms treated by a mixture of independent and constrained refinement
Δρ_max_ = 0.41 e Å^−3^
Δρ_min_ = −0.25 e Å^−3^

### 

Data collection: *APEX2* (Bruker, 2009 ▶); cell refinement: *SAINT* (Bruker, 2009 ▶); data reduction: *SAINT*; program(s) used to solve structure: *SHELXTL* (Sheldrick, 2008 ▶); program(s) used to refine structure: *SHELXTL*; molecular graphics: *SHELXTL*; software used to prepare material for publication: *SHELXTL* and *PLATON* (Spek, 2009 ▶).

## Supplementary Material

Crystal structure: contains datablock(s) global, I. DOI: 10.1107/S1600536811032673/tk2779sup1.cif

Structure factors: contains datablock(s) I. DOI: 10.1107/S1600536811032673/tk2779Isup2.hkl

Supplementary material file. DOI: 10.1107/S1600536811032673/tk2779Isup3.cml

Additional supplementary materials:  crystallographic information; 3D view; checkCIF report

## Figures and Tables

**Table 1:** Hydrogen-bond geometry (Å, °)

*D*—H⋯*A*	*D*—H	H⋯*A*	*D*⋯*A*	*D*—H⋯*A*
N3—H1*N*3⋯O1	0.90	2.39	2.9655 (11)	123
O1—H1*O*1⋯N2^i^	0.919 (16)	1.830 (16)	2.7038 (10)	158.0 (15)

Symmetry code: (i) 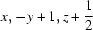.

**Fig. 1.**
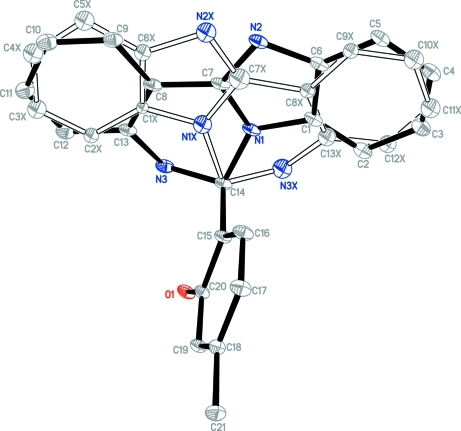
The molecular structure of (I), showing 50% probability displacement ellipsoids and the atom-numbering scheme. The minor component is shown with open bonds. H atoms omitted for clarity.

**Fig. 2.**
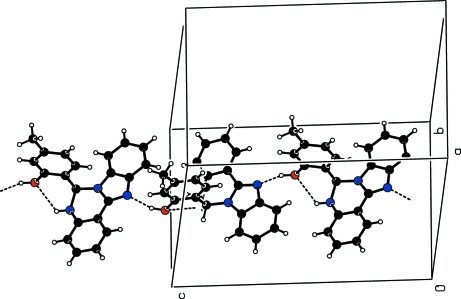
A view of the supramolecular chain in (I), dashed lines represents hydrogen bonding.

## supplementary crystallographic information

### Comment

Benzimidazoles are known to be strong chelating agents coordinating through both the C═N N atoms (Sun *et al.*, 2010). The benzimidazole ring system is present in the clinically approved anthelmintics, anti-ulcer, anti-viral, and anti-histamine drugs (Harrell *et al.*, 2004). As part of our on-going structural studies of benzimidazoles (Eltayeb *et al.*, 2007, 2009, 2011), we now describe in this paper the single-crystal X-ray diffraction study of title compound (I).

The molecular structure of (I) is shown in Fig. 1. The 2-(5,6 dihydrobenzimidazo[1,2-c]quinazolin-6-yl) molecule is disordered over two sites corresponding to a rotation of approximately 180\% with a refined occupancy ratio of 0.9180 (14):0.0820 (14). The imidazole (N1/N2/C6/C7/C8) ring is essentially planar, with a maximum deviation of 0.009 (1) Å for atom C7. The central pyrimidine ring makes dihedral angles of 5.02 (5), 3.97 (5), 6.28 (5) ° for the major component, and 5.8 (7), 5.0 (6) and 8.5 (6) ° for the minor component, respectively, with the plane of the imidazole and with those through the terminal phenyl rings.

In the crystal structure (Fig. 2), the molecules of the major component are connected by O1—H1O1···N2 hydrogen bonds (Table 1) forming a supramolecular chain along the *c*-axis.

### Experimental

To a solution of 2-(2-aminophenyl)-1*H*-benzimidazole (0.418 g, 2.0 mmol) in ethanol (30 mL) was added 2-hydroxy-4-methylbenzaldehyde (0.272 g, 2.0 mmol). The mixture was refluxed with stirring for two hours after which the colour of the resulting solution turned pale-yellow. Colourless crystals were formed after several days of slow evaporation of its ethanol solution held at room temperature.

### Refinement

Atom H1O1 was located from a difference Fourier maps and refined freely [O—H = 0.919 (16) Å]. The N—H H atoms were located from a difference map and fixed at those positions and refined with *U*_iso_(H) = 1.2 *U*_eq_(N). The remaining H atoms were positioned geometrically [C—H = 0.95–1.00 Å] and were refined using a riding model, with *U*_iso_(H) = 1.2 or 1.5 *U*_eq_(C). A rotating group model was applied to the methyl groups. The molecule is disordered over two sites with a refined occupancy ratio of 0.9180 (14):0.0820 (14); the minor component was refined with isotropic displacement parameters.

### Crystal data

**Table:**

C_21_H_17_N_3_O	*F*(000) = 1376
*M**_r_* = 327.38	*D*_x_ = 1.371 Mg m^−^^3^
Monoclinic, *C*2/*c*	Mo *K*α radiation, λ = 0.71073 Å
Hall symbol: -C 2yc	Cell parameters from 7250 reflections
*a* = 15.1292 (3) Å	θ = 2.4–33.4°
*b* = 12.2648 (2) Å	µ = 0.09 mm^−^^1^
*c* = 17.1909 (3) Å	*T* = 100 K
β = 96.233 (1)°	Block, colourless
*V* = 3171.03 (10) Å^3^	0.59 × 0.21 × 0.20 mm
*Z* = 8	

### Data collection

**Table:**

Bruker SMART APEXII CCD area-detector diffractometer	6164 independent reflections
Radiation source: fine-focus sealed tube	4808 reflections with *I* > 2σ(*I*)
graphite	*R*_int_ = 0.029
φ and ω scans	θ_max_ = 33.5°, θ_min_ = 2.1°
Absorption correction: multi-scan (*SADABS*; Bruker, 2009)	*h* = −23→21
*T*_min_ = 0.951, *T*_max_ = 0.983	*k* = −18→19
24277 measured reflections	*l* = −26→26

### Refinement

**Table:**

Refinement on *F*^2^	Primary atom site location: structure-invariant direct methods
Least-squares matrix: full	Secondary atom site location: difference Fourier map
*R*[*F*^2^ > 2σ(*F*^2^)] = 0.045	Hydrogen site location: inferred from neighbouring sites
*wR*(*F*^2^) = 0.126	H atoms treated by a mixture of independent and constrained refinement
*S* = 1.04	*w* = 1/[σ^2^(*F*_o_^2^) + (0.0635*P*)^2^ + 1.1622*P*] where *P* = (*F*_o_^2^ + 2*F*_c_^2^)/3
6164 reflections	(Δ/σ)_max_ = 0.001
296 parameters	Δρ_max_ = 0.41 e Å^−^^3^
44 restraints	Δρ_min_ = −0.25 e Å^−^^3^

### Special details

**Table:**

Experimental. The crystal was placed in the cold stream of an Oxford Cryosystems Cobra open-flow nitrogen cryostat (Cosier & Glazer, 1986) operating at 100.0 (1)K.
Geometry. All s.u.'s (except the s.u. in the dihedral angle between two l.s. planes) are estimated using the full covariance matrix. The cell s.u.'s are taken into account individually in the estimation of s.u.'s in distances, angles and torsion angles; correlations between s.u.'s in cell parameters are only used when they are defined by crystal symmetry. An approximate (isotropic) treatment of cell s.u.'s is used for estimating s.u.'s involving l.s. planes.
Refinement. Refinement of F^2^ against ALL reflections. The weighted R-factor wR and goodness of fit S are based on F^2^, conventional R-factors R are based on F, with F set to zero for negative F^2^. The threshold expression of F^2^ > 2σ(F^2^) is used only for calculating R-factors(gt) etc. and is not relevant to the choice of reflections for refinement. R-factors based on F^2^ are statistically about twice as large as those based on F, and R- factors based on ALL data will be even larger.

### Fractional atomic coordinates and isotropic or equivalent isotropic displacement parameters (Å^2^)

**Table:**

	*x*	*y*	*z*	*U*_iso_*/*U*_eq_	Occ. (<1)
O1	0.11427 (4)	0.39987 (6)	1.02884 (4)	0.02061 (14)	
C14	0.09940 (6)	0.43709 (8)	0.86934 (5)	0.01907 (17)	
H14A	0.0600	0.3742	0.8790	0.023*	0.9180 (14)
H14B	0.0558	0.4551	0.9038	0.023*	0.0820 (14)
C15	0.18959 (6)	0.41867 (7)	0.91603 (5)	0.01793 (16)	
C16	0.26904 (6)	0.41994 (8)	0.88195 (5)	0.02215 (18)	
H16A	0.2675	0.4300	0.8270	0.027*	
C17	0.35059 (6)	0.40677 (8)	0.92684 (5)	0.02231 (18)	
H17A	0.4040	0.4083	0.9025	0.027*	
C18	0.35418 (6)	0.39130 (7)	1.00739 (5)	0.01832 (16)	
C19	0.27492 (6)	0.38872 (7)	1.04197 (5)	0.01798 (16)	
H19A	0.2766	0.3778	1.0968	0.022*	
C20	0.19305 (5)	0.40201 (7)	0.99689 (5)	0.01667 (15)	
C21	0.44220 (6)	0.37331 (8)	1.05544 (5)	0.02230 (18)	
H21A	0.4406	0.4057	1.1074	0.033*	
H21B	0.4895	0.4076	1.0294	0.033*	
H21C	0.4537	0.2949	1.0608	0.033*	
N1	0.10675 (5)	0.44305 (7)	0.78585 (4)	0.01890 (16)	0.9180 (14)
N2	0.14473 (6)	0.52085 (8)	0.67606 (5)	0.02089 (17)	0.9180 (14)
N3	0.05600 (5)	0.53695 (7)	0.89063 (5)	0.02081 (17)	0.9180 (14)
H1N3	0.0569	0.5417	0.9427	0.031*	0.9180 (14)
C1	0.12136 (6)	0.35766 (10)	0.73616 (6)	0.01981 (19)	0.9180 (14)
C2	0.11768 (7)	0.24516 (11)	0.74523 (7)	0.0247 (2)	0.9180 (14)
H2A	0.1025	0.2128	0.7922	0.030*	0.9180 (14)
C3	0.13738 (8)	0.18239 (10)	0.68194 (8)	0.0260 (2)	0.9180 (14)
H3A	0.1354	0.1052	0.6855	0.031*	0.9180 (14)
C4	0.16018 (8)	0.23106 (12)	0.61273 (6)	0.0255 (2)	0.9180 (14)
H4A	0.1736	0.1858	0.5707	0.031*	0.9180 (14)
C5	0.16364 (7)	0.34331 (11)	0.60427 (6)	0.0238 (2)	0.9180 (14)
H5A	0.1787	0.3755	0.5572	0.029*	0.9180 (14)
C6	0.14422 (6)	0.40766 (9)	0.66750 (6)	0.02034 (19)	0.9180 (14)
C7	0.12315 (7)	0.53821 (11)	0.74761 (7)	0.01833 (19)	0.9180 (14)
C8	0.11421 (6)	0.64094 (9)	0.78768 (6)	0.01890 (18)	0.9180 (14)
C9	0.13375 (7)	0.74150 (10)	0.75533 (6)	0.0228 (2)	0.9180 (14)
H9A	0.1549	0.7441	0.7053	0.027*	0.9180 (14)
C10	0.12243 (8)	0.83764 (11)	0.79570 (8)	0.0267 (2)	0.9180 (14)
H10A	0.1356	0.9060	0.7736	0.032*	0.9180 (14)
C11	0.09131 (8)	0.83251 (12)	0.86945 (7)	0.0254 (2)	0.9180 (14)
H11A	0.0844	0.8979	0.8978	0.030*	0.9180 (14)
C12	0.07047 (7)	0.73363 (10)	0.90174 (6)	0.0229 (2)	0.9180 (14)
H12A	0.0484	0.7319	0.9514	0.027*	0.9180 (14)
C13	0.08176 (6)	0.63642 (9)	0.86160 (6)	0.01901 (18)	0.9180 (14)
N1X	0.1041 (7)	0.5258 (8)	0.8082 (6)	0.028 (2)*	0.0820 (14)
N2X	0.1354 (7)	0.6061 (8)	0.6958 (6)	0.029 (2)*	0.0820 (14)
N3X	0.0707 (8)	0.3422 (9)	0.8217 (6)	0.034 (3)*	0.0820 (14)
H3XB	0.0286	0.3007	0.8375	0.040*	0.0820 (14)
C1X	0.1029 (6)	0.6359 (7)	0.8222 (6)	0.0121 (17)*	0.0820 (14)
C2X	0.0875 (8)	0.6912 (10)	0.8879 (6)	0.019 (2)*	0.0820 (14)
H2XA	0.0751	0.6539	0.9339	0.023*	0.0820 (14)
C3X	0.0905 (11)	0.7992 (12)	0.8855 (9)	0.029 (4)*	0.0820 (14)
H3XA	0.0785	0.8413	0.9295	0.035*	0.0820 (14)
C4X	0.1115 (12)	0.8496 (12)	0.8177 (9)	0.030 (4)*	0.0820 (14)
H4XA	0.1149	0.9269	0.8181	0.035*	0.0820 (14)
C5X	0.1276 (9)	0.7977 (10)	0.7500 (7)	0.028 (3)*	0.0820 (14)
H5XA	0.1411	0.8352	0.7044	0.034*	0.0820 (14)
C6X	0.1223 (8)	0.6841 (9)	0.7548 (6)	0.021 (2)*	0.0820 (14)
C7X	0.1253 (14)	0.5126 (10)	0.7308 (9)	0.034 (5)*	0.0820 (14)
C8X	0.1320 (7)	0.3976 (8)	0.7053 (7)	0.0171 (19)*	0.0820 (14)
C9X	0.1614 (7)	0.3764 (9)	0.6341 (7)	0.017 (2)*	0.0820 (14)
H9XA	0.1765	0.4355	0.6025	0.021*	0.0820 (14)
C10X	0.1692 (11)	0.2743 (12)	0.6081 (8)	0.029 (3)*	0.0820 (14)
H10B	0.1906	0.2606	0.5591	0.034*	0.0820 (14)
C11X	0.1456 (11)	0.1902 (12)	0.6538 (9)	0.030 (4)*	0.0820 (14)
H11B	0.1510	0.1172	0.6365	0.036*	0.0820 (14)
C12X	0.1147 (9)	0.2095 (10)	0.7232 (8)	0.025 (3)*	0.0820 (14)
H12B	0.0983	0.1493	0.7534	0.031*	0.0820 (14)
C13X	0.1062 (9)	0.3127 (10)	0.7517 (7)	0.025 (2)*	0.0820 (14)
H1O1	0.1244 (10)	0.4082 (13)	1.0822 (10)	0.046 (4)*	

### Atomic displacement parameters (Å^2^)

**Table:**

	*U*^11^	*U*^22^	*U*^33^	*U*^12^	*U*^13^	*U*^23^
O1	0.0165 (3)	0.0344 (4)	0.0115 (3)	−0.0010 (2)	0.0041 (2)	−0.0009 (2)
C14	0.0158 (4)	0.0302 (4)	0.0115 (3)	−0.0020 (3)	0.0024 (3)	0.0028 (3)
C15	0.0160 (4)	0.0263 (4)	0.0116 (3)	−0.0006 (3)	0.0019 (3)	0.0011 (3)
C16	0.0182 (4)	0.0354 (5)	0.0132 (4)	−0.0006 (3)	0.0032 (3)	0.0027 (3)
C17	0.0163 (4)	0.0340 (5)	0.0172 (4)	0.0000 (3)	0.0042 (3)	0.0021 (3)
C18	0.0172 (4)	0.0218 (4)	0.0157 (4)	0.0005 (3)	0.0007 (3)	0.0002 (3)
C19	0.0176 (4)	0.0241 (4)	0.0121 (3)	0.0005 (3)	0.0011 (3)	0.0000 (3)
C20	0.0158 (3)	0.0221 (4)	0.0124 (3)	−0.0005 (3)	0.0031 (3)	−0.0009 (3)
C21	0.0180 (4)	0.0285 (4)	0.0199 (4)	0.0017 (3)	−0.0004 (3)	0.0023 (3)
N1	0.0170 (3)	0.0294 (4)	0.0104 (3)	−0.0026 (3)	0.0021 (2)	0.0019 (3)
N2	0.0194 (4)	0.0322 (4)	0.0114 (3)	0.0007 (3)	0.0031 (3)	0.0018 (3)
N3	0.0171 (4)	0.0327 (4)	0.0135 (3)	0.0030 (3)	0.0060 (3)	0.0039 (3)
C1	0.0157 (4)	0.0308 (5)	0.0127 (4)	−0.0031 (4)	0.0006 (3)	−0.0001 (4)
C2	0.0242 (5)	0.0323 (6)	0.0174 (5)	−0.0038 (4)	0.0020 (4)	−0.0006 (4)
C3	0.0249 (5)	0.0312 (6)	0.0214 (6)	−0.0048 (4)	0.0012 (4)	−0.0016 (4)
C4	0.0237 (5)	0.0340 (7)	0.0182 (5)	−0.0030 (5)	0.0002 (3)	−0.0039 (4)
C5	0.0229 (5)	0.0349 (6)	0.0138 (4)	−0.0010 (4)	0.0023 (3)	−0.0020 (4)
C6	0.0167 (4)	0.0323 (5)	0.0118 (4)	−0.0013 (4)	0.0007 (3)	0.0000 (4)
C7	0.0127 (4)	0.0315 (5)	0.0108 (4)	−0.0006 (4)	0.0015 (3)	0.0033 (4)
C8	0.0146 (4)	0.0301 (5)	0.0122 (4)	0.0028 (3)	0.0027 (3)	0.0034 (3)
C9	0.0219 (5)	0.0298 (6)	0.0177 (4)	0.0039 (4)	0.0060 (3)	0.0051 (4)
C10	0.0266 (6)	0.0327 (6)	0.0222 (6)	0.0056 (4)	0.0093 (4)	0.0059 (5)
C11	0.0262 (5)	0.0291 (6)	0.0220 (5)	0.0065 (5)	0.0075 (4)	0.0021 (5)
C12	0.0208 (4)	0.0316 (5)	0.0170 (4)	0.0062 (4)	0.0050 (3)	0.0013 (4)
C13	0.0136 (4)	0.0312 (5)	0.0124 (4)	0.0031 (3)	0.0021 (3)	0.0030 (3)

### Geometric parameters (Å, °)

**Table:**

O1—C20	1.3658 (10)	C7—C8	1.4493 (18)
O1—H1O1	0.919 (16)	C8—C9	1.3976 (15)
C14—N1	1.4534 (11)	C8—C13	1.4122 (13)
C14—N3	1.4551 (13)	C9—C10	1.3883 (19)
C14—N3X	1.462 (12)	C9—H9A	0.9500
C14—N1X	1.520 (10)	C10—C11	1.4010 (17)
C14—C15	1.5230 (12)	C10—H10A	0.9500
C14—H14A	1.0000	C11—C12	1.3842 (17)
C14—H14B	0.9600	C11—H11A	0.9500
C15—C16	1.3932 (12)	C12—C13	1.3973 (15)
C15—C20	1.4003 (11)	C12—H12A	0.9500
C16—C17	1.3918 (13)	N1X—C1X	1.372 (11)
C16—H16A	0.9500	N1X—C7X	1.411 (14)
C17—C18	1.3928 (12)	N2X—C7X	1.310 (14)
C17—H17A	0.9500	N2X—C6X	1.424 (12)
C18—C19	1.3955 (12)	N3X—C13X	1.416 (13)
C18—C21	1.5052 (12)	N3X—H3XB	0.8800
C19—C20	1.3974 (11)	C1X—C6X	1.360 (13)
C19—H19A	0.9500	C1X—C2X	1.360 (13)
C21—H21A	0.9800	C2X—C3X	1.326 (14)
C21—H21B	0.9800	C2X—H2XA	0.9500
C21—H21C	0.9800	C3X—C4X	1.386 (15)
N1—C7	1.3752 (14)	C3X—H3XA	0.9500
N1—C1	1.3843 (13)	C4X—C5X	1.371 (14)
N2—C7	1.3236 (14)	C4X—H4XA	0.9500
N2—C6	1.3959 (14)	C5X—C6X	1.399 (14)
N3—C13	1.3898 (13)	C5X—H5XA	0.9500
N3—H14B	1.0293	C7X—C8X	1.484 (13)
N3—H1N3	0.8957	C8X—C9X	1.372 (13)
C1—C2	1.3904 (17)	C8X—C13X	1.394 (14)
C1—C6	1.4063 (14)	C9X—C10X	1.339 (14)
C2—C3	1.3912 (17)	C9X—H9XA	0.9500
C2—H2A	0.9500	C10X—C11X	1.367 (15)
C3—C4	1.4071 (17)	C10X—H10B	0.9500
C3—H3A	0.9500	C11X—C12X	1.349 (14)
C4—C5	1.3860 (18)	C11X—H11B	0.9500
C4—H4A	0.9500	C12X—C13X	1.368 (14)
C5—C6	1.3999 (14)	C12X—H12B	0.9500
C5—H5A	0.9500		
			
C20—O1—H1O1	109.9 (10)	N2—C6—C1	110.13 (9)
N1—C14—N3	106.92 (7)	C5—C6—C1	119.82 (10)
N1—C14—N3X	62.4 (4)	N2—C7—N1	112.53 (12)
N3—C14—N3X	134.1 (5)	N2—C7—C8	128.81 (10)
N3—C14—N1X	68.1 (4)	N1—C7—C8	118.66 (10)
N3X—C14—N1X	102.5 (6)	C9—C8—C13	120.12 (10)
N1—C14—C15	111.57 (7)	C9—C8—C7	122.91 (10)
N3—C14—C15	113.28 (7)	C13—C8—C7	116.94 (9)
N3X—C14—C15	111.8 (5)	C10—C9—C8	120.51 (10)
N1X—C14—C15	111.4 (4)	C10—C9—H9A	119.7
N1—C14—H14A	108.3	C8—C9—H9A	119.7
N3—C14—H14A	108.3	C9—C10—C11	119.08 (12)
N3X—C14—H14A	48.1	C9—C10—H10A	120.5
N1X—C14—H14A	137.8	C11—C10—H10A	120.5
C15—C14—H14A	108.3	C12—C11—C10	121.04 (12)
N1—C14—H14B	136.9	C12—C11—H11A	119.5
N3X—C14—H14B	110.3	C10—C11—H11A	119.5
N1X—C14—H14B	110.5	C11—C12—C13	120.28 (9)
C15—C14—H14B	110.2	C11—C12—H12A	119.9
H14A—C14—H14B	67.2	C13—C12—H12A	119.9
C16—C15—C20	118.56 (8)	N3—C13—C12	121.17 (9)
C16—C15—C14	122.77 (7)	N3—C13—C8	119.74 (9)
C20—C15—C14	118.66 (7)	C12—C13—C8	118.94 (9)
C17—C16—C15	121.26 (8)	C1X—N1X—C7X	106.7 (9)
C17—C16—H16A	119.4	C1X—N1X—C14	125.5 (8)
C15—C16—H16A	119.4	C7X—N1X—C14	127.1 (8)
C16—C17—C18	120.23 (8)	C7X—N2X—C6X	103.3 (9)
C16—C17—H17A	119.9	C13X—N3X—C14	124.2 (10)
C18—C17—H17A	119.9	C13X—N3X—H3XB	117.9
C17—C18—C19	118.96 (8)	C14—N3X—H3XB	117.9
C17—C18—C21	120.15 (8)	C6X—C1X—C2X	124.2 (9)
C19—C18—C21	120.85 (8)	C6X—C1X—N1X	105.7 (9)
C18—C19—C20	120.79 (8)	C2X—C1X—N1X	130.1 (10)
C18—C19—H19A	119.6	C3X—C2X—C1X	117.6 (10)
C20—C19—H19A	119.6	C3X—C2X—H2XA	121.2
O1—C20—C19	122.30 (7)	C1X—C2X—H2XA	121.2
O1—C20—C15	117.50 (7)	C2X—C3X—C4X	118.9 (13)
C19—C20—C15	120.19 (8)	C2X—C3X—H3XA	120.5
C18—C21—H21A	109.5	C4X—C3X—H3XA	120.5
C18—C21—H21B	109.5	C5X—C4X—C3X	125.8 (14)
H21A—C21—H21B	109.5	C5X—C4X—H4XA	117.1
C18—C21—H21C	109.5	C3X—C4X—H4XA	117.1
H21A—C21—H21C	109.5	C4X—C5X—C6X	113.3 (12)
H21B—C21—H21C	109.5	C4X—C5X—H5XA	123.4
C7—N1—C1	107.34 (9)	C6X—C5X—H5XA	123.4
C7—N1—C14	123.50 (9)	C1X—C6X—C5X	120.2 (9)
C1—N1—C14	127.28 (9)	C1X—C6X—N2X	111.9 (9)
C7—N2—C6	105.01 (9)	C5X—C6X—N2X	127.9 (10)
C13—N3—C14	119.79 (8)	N2X—C7X—N1X	112.5 (10)
C13—N3—H14B	160.6	N2X—C7X—C8X	132.9 (12)
C13—N3—H1N3	109.1	N1X—C7X—C8X	114.6 (11)
C14—N3—H1N3	110.4	C9X—C8X—C13X	120.4 (9)
H14B—N3—H1N3	81.0	C9X—C8X—C7X	118.9 (10)
N1—C1—C2	132.12 (10)	C13X—C8X—C7X	120.6 (10)
N1—C1—C6	104.97 (10)	C10X—C9X—C8X	121.7 (10)
C2—C1—C6	122.91 (10)	C10X—C9X—H9XA	119.2
C1—C2—C3	116.55 (10)	C8X—C9X—H9XA	119.2
C1—C2—H2A	121.7	C9X—C10X—C11X	118.3 (12)
C3—C2—H2A	121.7	C9X—C10X—H10B	120.8
C2—C3—C4	121.30 (12)	C11X—C10X—H10B	120.8
C2—C3—H3A	119.4	C12X—C11X—C10X	120.9 (13)
C4—C3—H3A	119.4	C12X—C11X—H11B	119.5
C5—C4—C3	121.71 (11)	C10X—C11X—H11B	119.5
C5—C4—H4A	119.1	C11X—C12X—C13X	122.3 (12)
C3—C4—H4A	119.1	C11X—C12X—H12B	118.8
C4—C5—C6	117.71 (10)	C13X—C12X—H12B	118.8
C4—C5—H5A	121.1	C12X—C13X—C8X	116.3 (10)
C6—C5—H5A	121.1	C12X—C13X—N3X	126.9 (11)
N2—C6—C5	130.04 (10)	C8X—C13X—N3X	116.8 (10)
			
N1—C14—C15—C16	6.57 (13)	C9—C10—C11—C12	−1.08 (18)
N3—C14—C15—C16	−114.14 (10)	C10—C11—C12—C13	1.18 (17)
N3X—C14—C15—C16	74.4 (5)	C14—N3—C13—C12	−154.10 (9)
N1X—C14—C15—C16	−39.6 (4)	C14—N3—C13—C8	30.38 (13)
N1—C14—C15—C20	−174.80 (8)	C11—C12—C13—N3	−175.88 (10)
N3—C14—C15—C20	64.49 (11)	C11—C12—C13—C8	−0.33 (15)
N3X—C14—C15—C20	−107.0 (5)	C9—C8—C13—N3	175.03 (9)
N1X—C14—C15—C20	139.0 (4)	C7—C8—C13—N3	−3.20 (13)
C20—C15—C16—C17	−1.02 (14)	C9—C8—C13—C12	−0.59 (14)
C14—C15—C16—C17	177.62 (9)	C7—C8—C13—C12	−178.82 (9)
C15—C16—C17—C18	0.38 (15)	N1—C14—N1X—C1X	−179.2 (14)
C16—C17—C18—C19	0.31 (14)	N3—C14—N1X—C1X	27.4 (9)
C16—C17—C18—C21	177.94 (9)	N3X—C14—N1X—C1X	160.2 (10)
C17—C18—C19—C20	−0.36 (13)	C15—C14—N1X—C1X	−80.1 (10)
C21—C18—C19—C20	−177.97 (8)	N1—C14—N1X—C7X	−10.8 (12)
C18—C19—C20—O1	179.97 (8)	N3—C14—N1X—C7X	−164.3 (15)
C18—C19—C20—C15	−0.29 (13)	N3X—C14—N1X—C7X	−31.4 (15)
C16—C15—C20—O1	−179.28 (8)	C15—C14—N1X—C7X	88.3 (14)
C14—C15—C20—O1	2.03 (12)	N1—C14—N3X—C13X	28.9 (9)
C16—C15—C20—C19	0.97 (13)	N3—C14—N3X—C13X	116.0 (10)
C14—C15—C20—C19	−177.72 (8)	N1X—C14—N3X—C13X	44.5 (12)
N3—C14—N1—C7	36.05 (12)	C15—C14—N3X—C13X	−74.9 (11)
N3X—C14—N1—C7	167.5 (5)	C7X—N1X—C1X—C6X	0.4 (14)
N1X—C14—N1—C7	10.3 (6)	C14—N1X—C1X—C6X	170.7 (9)
C15—C14—N1—C7	−88.30 (11)	C7X—N1X—C1X—C2X	−178.8 (13)
N3—C14—N1—C1	−161.62 (9)	C14—N1X—C1X—C2X	−8.5 (18)
N3X—C14—N1—C1	−30.1 (5)	C6X—C1X—C2X—C3X	0.9 (18)
N1X—C14—N1—C1	172.6 (6)	N1X—C1X—C2X—C3X	180.0 (13)
C15—C14—N1—C1	74.02 (11)	C1X—C2X—C3X—C4X	−2(2)
N1—C14—N3—C13	−43.94 (11)	C2X—C3X—C4X—C5X	2(3)
N3X—C14—N3—C13	−111.7 (6)	C3X—C4X—C5X—C6X	−1(2)
N1X—C14—N3—C13	−25.4 (4)	C2X—C1X—C6X—C5X	0.1 (18)
C15—C14—N3—C13	79.37 (10)	N1X—C1X—C6X—C5X	−179.1 (11)
C7—N1—C1—C2	177.61 (11)	C2X—C1X—C6X—N2X	179.9 (10)
C14—N1—C1—C2	12.99 (17)	N1X—C1X—C6X—N2X	0.6 (13)
C7—N1—C1—C6	−1.60 (10)	C4X—C5X—C6X—C1X	−0.2 (19)
C14—N1—C1—C6	−166.22 (8)	C4X—C5X—C6X—N2X	−179.9 (13)
N1—C1—C2—C3	−179.53 (10)	C7X—N2X—C6X—C1X	−1.4 (16)
C6—C1—C2—C3	−0.44 (15)	C7X—N2X—C6X—C5X	178.3 (15)
C1—C2—C3—C4	0.29 (16)	C6X—N2X—C7X—N1X	1.6 (19)
C2—C3—C4—C5	−0.32 (17)	C6X—N2X—C7X—C8X	−178.1 (19)
C3—C4—C5—C6	0.48 (16)	C1X—N1X—C7X—N2X	−1.4 (19)
C7—N2—C6—C5	−178.71 (10)	C14—N1X—C7X—N2X	−171.5 (11)
C7—N2—C6—C1	0.18 (11)	C1X—N1X—C7X—C8X	178.5 (13)
C4—C5—C6—N2	178.19 (10)	C14—N1X—C7X—C8X	8(2)
C4—C5—C6—C1	−0.61 (14)	N2X—C7X—C8X—C9X	6(3)
N1—C1—C6—N2	0.90 (10)	N1X—C7X—C8X—C9X	−173.3 (13)
C2—C1—C6—N2	−178.40 (9)	N2X—C7X—C8X—C13X	−171.4 (19)
N1—C1—C6—C5	179.92 (9)	N1X—C7X—C8X—C13X	9(2)
C2—C1—C6—C5	0.62 (15)	C13X—C8X—C9X—C10X	−2.4 (18)
C6—N2—C7—N1	−1.25 (11)	C7X—C8X—C9X—C10X	179.7 (14)
C6—N2—C7—C8	179.60 (10)	C8X—C9X—C10X—C11X	1(2)
C1—N1—C7—N2	1.86 (12)	C9X—C10X—C11X—C12X	0(2)
C14—N1—C7—N2	167.20 (8)	C10X—C11X—C12X—C13X	−1(2)
C1—N1—C7—C8	−178.89 (9)	C11X—C12X—C13X—C8X	−1(2)
C14—N1—C7—C8	−13.55 (14)	C11X—C12X—C13X—N3X	176.7 (14)
N2—C7—C8—C9	−4.22 (17)	C9X—C8X—C13X—C12X	2.0 (18)
N1—C7—C8—C9	176.67 (9)	C7X—C8X—C13X—C12X	179.9 (14)
N2—C7—C8—C13	173.96 (10)	C9X—C8X—C13X—N3X	−175.5 (11)
N1—C7—C8—C13	−5.15 (14)	C7X—C8X—C13X—N3X	2.3 (19)
C13—C8—C9—C10	0.68 (15)	C14—N3X—C13X—C12X	148.8 (13)
C7—C8—C9—C10	178.80 (10)	C14—N3X—C13X—C8X	−33.9 (17)
C8—C9—C10—C11	0.14 (17)		

### Hydrogen-bond geometry (Å, °)

**Table:**

*D*—H···*A*	*D*—H	H···*A*	*D*···*A*	*D*—H···*A*
N3—H1N3···O1	0.90	2.39	2.9655 (11)	123
O1—H1O1···N2^i^	0.919 (16)	1.830 (16)	2.7038 (10)	158.0 (15)

Symmetry codes: (i) *x*, −*y*+1, *z*+1/2.
